# Analysis of the anatomical distribution of HPV genotypes in head and neck squamous papillomas

**DOI:** 10.1371/journal.pone.0290004

**Published:** 2023-08-11

**Authors:** Huiying Hu, Huanyu Jiang, Zhenwen Zhu, Honglin Yin, Kai Liu, Lijuan Chen, Mengyuan Zhao, Zhenkun Yu

**Affiliations:** 1 School of Medicine, Southeast University, Nanjing, China; 2 Department of Otolaryngology Head and Neck Surgery, The Affiliated BenQ Hospital of Nanjing Medical University, Nanjing, China; 3 Department of Pathology, The Affiliated BenQ Hospital of Nanjing Medical University, Nanjing, China; Marche University Hospital: Azienda Ospedaliero Universitaria delle Marche, ITALY

## Abstract

Squamous papillomas (SPs) of the head and neck are usually benign lesions associated with human papilloma virus (HPV) infection. However, the reported HPV detection rates vary widely, especially with respect to anatomical distribution. The etiology of SPs in the head and neck remains unclear; analyzing HPV genotypes of SPs based on anatomical sites could assist in clarifying the pathogenesis of SPs in the head and neck. Therefore, the aim of this study was to review the prevalence, subtypes, and anatomical distribution of HPV in head and neck SPs at a hospital in China; we also investigated whether p16, a marker of HPV infection in oropharyngeal carcinoma, could serve as a surrogate marker for HPV in head and neck SPs. The presence of HPV DNA of 23 types (5 low-risk HPV and 18 high-risk HPV types) was detected via real-time PCR. p16 immunohistochemistry was performed using SP sections. Age, sex, anatomical location, and HPV subtype were recorded for each case. In total, 105 SPs were identified, including 47 in the larynx, 42 in the pharynx, 6 in the external auditory canal (EAC), 5 in the oral cavity, and 5 in the nasal cavity. HPV was found in 57 (54.3%) cases, with the highest positivity rate in the larynx (46/47; 97.9%). Only 5/42 (11.9%) patients showed HPV positivity in the pharynx. HPV incidence was highly dependent on the anatomical site. SPs in the larynx and EAC were more likely to carry HPV than those in other anatomical sites. High-risk HPV infections were rarely associated with SPs in the head and neck region. The sensitivity and specificity of p16 immunohistochemistry for HPV infection were 88% and 96%, respectively. There may be an association between p16 and HPV infection in head and neck SPs, but further studies are needed to validate this assertion.

## Introduction

Squamous papillomas (SPs) are histologically benign growths typically encountered from the nasal vestibule to the respiratory tract in patients visiting an otolaryngology department. SPs are precancerous lesions that can occasionally cause airway obstruction [1]. The etiology of SPs in the head and neck has not been universally confirmed, vast variation in the published report of HPV status has resulted in widely disparate views of the role of HPV as a pathogenic agent in SP [2, 3]. HPV DNA is subdivided into low-risk HPV (LR-HPV) and high-risk HPV (HR-HPV) types, based on tropism and the ability to cause cancer. The association of HPV with oropharyngeal carcinoma [4] and recurrent respiratory papillomatosis (RRP) is a major concern; however, HPV is less frequently associated with other SPs in the head and neck [5–7]. We hypothesized that the HPV infection rate is related to the different anatomical sites of SPs.

HPV genotyping for papilloma is not routinely performed in otorhinolaryngology clinics. Although usually benign, SPs can clinically show widespread growth and recurrence, and some tend to be malignant, with a possibility of HPV involvement. Analyzing the HPV genotypes of SPs based on anatomical sites could contribute to our understanding of the pathogenesis of SPs in the head and neck. Therefore, in this study, we aimed to investigate the existence, subtypes, and anatomical distribution of HPV in head and neck SPs in a Chinese population; we also aimed to establish whether p16 can serve as a surrogate marker for HPV infection in head and neck SPs.

## Materials and methods

### Ethics approval

The protocols for this study were approved by the Institutional Review Board of BenQ Hospital Affiliated with Nanjing Medical University (Decision No. 2020-KL013). We obtained written informed consent from all participants in the study and the consent of parents or guardians of minors before starting the study. All of the procedures were performed in accordance with the Declaration of Helsinki and relevant policies in China.

### Patient samples and clinical data

Our study comprised 105 patients diagnosed with SPs, selected from a total of 8063 patients who underwent surgical treatment at the Department of Otorhinolaryngology Head and Neck Surgery of the affiliated BenQ Hospital of Nanjing Medical University from July 2018 to December 2022. All cases diagnosed with SPs in head and neck were identified through a search of the pathology files, and lesions mostly came from mucosal membranes and the external auditory canal (EAC) skin excluding surrounding skin warts. They were reviewed from December 2020 to December 2022. The histological diagnosis was confirmed by an experienced head and neck pathologist. Inclusion criteria comprehended: (a) diagnosis of SPs; (b) anatomic site was in head and neck; (c) surgical treatment; (d) histological and clinical data available in hospital database. Final pathological diagnoses of cancer were excluded.

Medical records were retrospectively evaluated for age, sex, clinical presentation, otoscopic findings, previous treatments, surgical findings, histopathology, follow-up intervals, and recurrence. Cases of recurrence were defined as the reappearance of SPs at the identical anatomic location. This was evaluated by two seasoned pathologists. Authors had not access to information that could identify individual participants during or after data collection.

### HPV DNA detection and genotyping

Surgical tissue specimens were used for HPV DNA extraction. The surgical specimens were selected from 0.2 mm tumor-tissue blocks and placed in a special cell-preservation solution in a sampling tube marked with the patient’s barcode. All the samples were stored at 4°C and tested within 48 h. After DNA extraction, HPV DNA was hybridized using a Human Papillomavirus Genotyping Kit for 23 HPV types (PCR-RDB, Reverse Dot Blot, Yaneng BIO, Guangdong, China), including 17 high-risk types (HPV16, 18, 31, 33, 35, 39, 45, 51, 52, 56, 58, 59, 66, 68, 53,73, 82) and 6 low-risk types (HPV 6, 11, 42, 43, 44, 81). HPV DNA extraction, PCR amplification, hybridization, and interpretation of results were performed according to the manufacturer’s instructions.

### p16 immunohistochemistry

Initially identified SPs were selected for p16 immunohistochemistry [4]. SP blocks were cut to a thickness of 4 μm (microtome RM 2145; Leica Biosystems, Nussloch, Germany), deparaffinized, and treated with 10 mM citrate buffer at 92°C for 30 min for antigen retrieval. Slides were immunostained using a mouse monoclonal anti-p16 antibody (Jiangsu Qiming Gene Technology Company, China, ready-to-use antibody). p16 expression was rated by two pathologists who were blinded to the patients’ details. Staining was performed in the presence of appropriate controls according to the manufacturer’s instructions. According to the fraction of positive cells, the degree of p16 expression was determined: negative staining = 0, 1–5% = 1, 6–25% = 2, 26–75% = 3, >75% = 4.

### Statistical analysis

All statistical analyses were performed using SPSS software (version 24.0; IBM Corp., Armonk, NY, USA). For continuous variables, the mean and standard deviation were used for statistical analyses. The chi-square test, t-test, and rank sum test were used for hypothesis testing, and *p* < 0.05 was considered statistically significant.

## Results

### Demographics

In total, 105 SPs were included in this analysis; 47 papillomas were identified in the larynx as recurrent respiratory papillomatosis, 42 were identified in the pharynx, 6 were identified in the EAC, 5 were identified in the oral cavity, and 5 were identified in the nasal cavity in both adults and children. The prevalence of SPs is high among males, as 77 (73.3%) patients diagnosed with SPs were male. The mean age at the time of surgery was 35.1 years (range, 0.6–83 years). The mean follow-up time after primary surgical resection was 22.9 months (range, 1–46 months) (Table 1).

**Table 1:** Clinical and demographic information.

Region	No.	Age (mean ± SD)	Male, N (%)	Recurrence rate, N (%)
**Larynx**	47	18.16±16.39	32 (68.1)	22 (46.8) ^a^
**Pharynx**	42	47.39±15.34	33 (78.6)	0 (0.0)
**EAC**	6	58.00±15.75	6 (100)	1 (16.7) ^a^
**Oral cavity**	5	50.60±12.58	3 (60%)	0 (0.0)
**Nasal cavity**	5	48.00±10.23	3 (60.0)	0 (0.0)
**Overall**	105	35.10±21.77	77 (73.3)	23 (21.9)

^a^ Differences between the marked groups were statistically significant.

### Clinical symptoms of papilloma in different regions

The tonsils were most commonly affected by oropharyngeal papillomas. Most patients had no symptoms, and papillomas were discovered inadvertently during laryngoscopy or gastroscopy; RRP was accompanied by clinical manifestations of hoarseness and dyspnea. The clinical presentation of EAC papilloma usually involved a mass in the EAC, bleeding, aural fullness, or hearing loss. The clinical presentation of nasal papilloma was usually a mass in the nose with bleeding. The anatomic site exhibiting the most frequent recurrence of SPs in head and neck was in the larynx (22/47, 46.8%).

### HPV genotypes

HPV was found in 57 (54.3%) cases of SPs, frequently associated with the EAC and larynx regions (100%, and 97.9%, respectively), but not the pharynx (11.9%). The larynx group showed the highest rate of HPV11; the EAC and pharynx groups showed the lowest rates of HPV11, and the difference between the larynx and pharynx groups was statistically significant. The larynx group showed a significantly higher rate of HPV6, compared with that in the other groups. The larynx group exhibited the highest HPV positivity rate, whereas the pharynx group demonstrated the lowest rate. This disparity was statistically significant. (*p* < 0.001). Only laryngeal papillomas showed combinations of subtypes. Papillomas in the EAC were produced by infection with HPV6 (100%). HPV incidence was highly dependent on the anatomical site; HPV positive SPs mainly arising in the larynx than other anatomical sites. HR-HPV infections appeared to be rare, compared with LR-HPV infections of head and neck SPs (0.01%, *p* < 0.001) (Table 2).

**Table 2:** HPV status in papilloma tissue based on anatomical regions.

	HPV-positive papillomas, n (%)					
HPV genotype	Larynx n = 47	Pharynx n = 42	EAC n = 6	Oral cavity n = 5	Nasal cavity n = 5	All sites n = 105
**Low-risk**	46 (97.9)	4 (9.5)	6 (100)	0 (0.0)	0 (0.0)	56 (53.3)
**11**	25 (53.2)	2 (4.8)	0 (0.0)	-	-	27 (25.7)
**6**	17 (36.2)	2 (4.8)	6 (100)	-	-	25 (23.8)
**6+11**	3 (6.4)	0 (0.0)	0 (0.0)	-	-	3 (2.9)
**6+42**	1 (2.1)	0 (0.0)	0 (0.0)	-	-	1 (0.01)
**High-risk**	0 (0.0)	1 (2.4)	0 (0.0)	0 (0.0)	0 (0.0)	1 (0.01)
**16**	-	1 (2.4)	-	-	-	1 (0.01)
**Overall**	46 (97.9)	5 (11.9)	6 (100)	0 (0.0)	0 (0.0)	57 (54.3)

EAC, external auditory canal

A total of 46 cases (46/47, 97.8%) of laryngeal papillomas were positive for HPV, 4/47 (8.5%) showed multiple HPV types. Among the cases in which various types of HPV were detected, 25/47 (53.2%) were positive for HPV11 and 17/47 (36.2%) were positive for HPV6. The present study found no statistically significant difference in the HPV positive rate between two distinct age groups: juvenile RRP (JO-RRP, < 18 years old) and adult RRP (AO-RRP, ≥18 years old) (Table 3).

**Table 3:** HPV genotypes in recurrent respiratory papillomatosis (RRP).

HPV genotype, n (%)						
	No. of cases	HPV11	HPV6	HPV11+6	HPV6+42	Overall
**JO-RRP**	28	15 (53.6)	10 (35.7)	3 (10.7)	0 (0.0)	28 (100)
**AO-RRP**	19	10 (50.0)	7 (36.8)	0 (0.0)	1 (5.3)	18 (94.7)
**Overall**	47	25 (53.1)	17 (36.2)	3 (6.4)	1 (2.1)	46 (97.3)

HPV, human papillomavirus; AO-RRP, adult patients (≥ 18 years of age) with RRP; JO-RRP, juvenile patients (<18 years of age) with RRP

### p16 Immunohistochemistry

**Fig 1:**
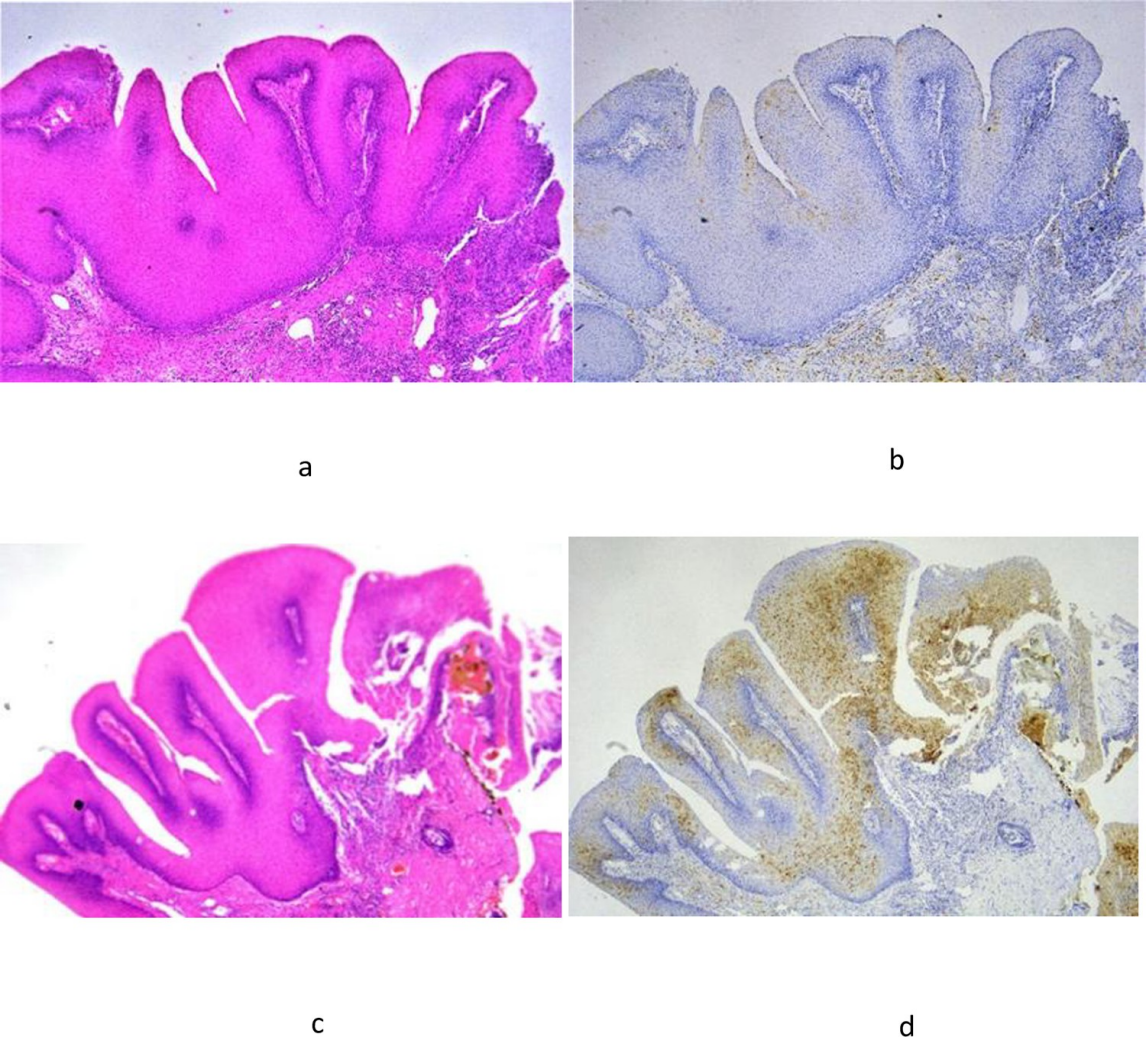
p16 staining of squamous papilloma samples. (a) Case 1 (HPV negative), squamous cell papilloma HE (40×). (b) Case 1 (HPV negative), negative for p16 IHC (40×). (c) Case 2 (HPV11 positive), squamous cell papilloma HE (40×). (d) Case 2 (HPV11 positive), positive for p16 IHC (40×).

SP samples containing HPV DNA also expressed p16. We stained 105 SP samples for p16 protein. In this sample set, 57 samples were HPV positive. Of these, 50 positively expressed p16 (Fig 1), and only 7 were p16 negative. Weak p16 staining (1+) was also observed in the two samples without HPV infection. In summary, the rate of false-positive cases was 4.2% (2/48), and 12.3% of the tumor samples (7/57) were false negatives. A significant correlation was observed between the degree of p16 IHC expression (degrees 1–3) and HPV-PCR results (Table 4). The sensitivity of p16 IHC for HPV infection was 88%, and the specificity was 96% (Table 5).

**Table 4:** Comparison of HPV DNA detection using PCR and p16 (IHC staining).

Degree of p16 expression	HPV DNA+	HPV DNA-	n
**0**	7	46	53
**1**	15	2	17
**2**	27	0	27
**3**	6	0	6
**4**	1	0	1
**n**	57	48	105

**Table 5:** Association of p16 (IHC) and HPV (PCR) in SPs.

	HPV+	HPV-	n	Sensitivity	Specificity
**p16+**	50	2	52	88%	96%
**p16-**	7	46	53		
**n**	57	48	105		

## Discussion

Although the association between HPV infection and the occurrence and progression of laryngeal papilloma and oropharyngeal carcinoma has been well documented, few studies have considered the variability of anatomical regions in the head and neck, and ambiguities continue to exist regarding HPV infection and the occurrence of SPs in the head and neck [8–10]. Here, we analyzed the HPV gene-type differences in 105 cases of SPs according to head and neck regions, in order to clarify the extent to which HPV plays a role in the occurrence of SPs in these lesions.

The results of associations between HPV and SPs in the literature are diverse, owing to different detection methods and the wide range of prevalence [2]. The sensitivity of HPV DNA detection has improved with real-time PCR. In our study, 46 cases of RRP (97.6%) were HPV positivity. The sensitivity of HPV DNA detection in RRP has improved with real-time PCR, and the HPV infection rate in RRP was recently found to be close to 100% [11–13]. HPV6 and HPV11 remained the two most predominant genotypes, and there was no co-infection with HR-HPV in our cases. The most frequent type was LR-HPV11, which represents the most prevalent HPV in laryngeal lesions, and HPV11 was found to be more prevalent in children than in adults [14]. It seems that children are more prone to compound infection and 3/28 (10.8%) cases were positive for multiple HPV types among our JO-RRP cases, which may explain the high recurrence rate in children with severe symptoms.

SPs in the pharynx are accidentally diagnosed, as most SPs are asymptomatic [6] and large case numbers are rarely documented. We detected 42 cases in 11.9% (5/42) of HPV cases and did not find HPV6/11 co-infections. This is consistent with the findings of published studies in Europe [2], the US [5], and Japan [11, 15]. We identified a positive case of HPV16 in the pharyngeal SPs, which was confirmed by positive p16 immunohistochemistry. Further follow-up and observation may be necessary for this case. In summary, the HPV positivity rate was low in the pharynx and mainly in LR-HPV infection. Considering the studies identifying HR-HPV in SPs [2, 16, 17], this association may drive the growing clinical practice of testing benign SPs for the presence of HR- HPV [5].

Papillomas involving the EAC are very rare, with only a few reports describing its involvement [18]. We confirmed the presence of the LR-HPV genotype HPV6 in EAC (6/6, 100%), implicating HPV6 in the etiology of EAC papillomas [19] in a small number of cases. HPV6-positive lesions may have resulted from contact with infectious agents via contaminated fingertips or ear-picking tools [19]. The findings of this study significantly contribute to the knowledge of the EAC. One limitation of our study was the small sample size and the fact that it was conducted at a single center. Additionally, the sample size in oral and nasal cavities was also limited, and the test results was negative. Further research is necessary to explore the potential benefits of increasing sample size and implementing multicenter studies. Protein p16 is a common tumor suppressor gene [5], and its protein expression has been demonstrated to be a reliable alternative marker of HPV infection in oropharyngeal carcinoma [6]; however, it is unclear whether p16 can also be used as a substitute marker in SPs. Determining the correlation between this marker and HPV in SPs could assist in providing a simple approach for identifying HPV infection in SPs. The results of HPV-PCR and p16-IHC staining were found to be significantly correlated in our study. These results are consistent with those of Ciesielska et al. [17]. In our study, p16 was significantly correlated with HPV positivity in SPs but not with anatomical sites or HPV DNA type. Thus, p16 may be used as a supplementary marker of HPV infection in SPs, further studies are needed to validate this assertion.

Vaccination against HPV can prevent HPV infection, but only two RRP patients were vaccinated in our cases with recurrences. Given the low coverage of HPV vaccines with the large population of this country, the limited health services in developing countries, high costs, and policy limits for the HPV vaccine [20], we did not examine the impact of the vaccine on the results of our study.

In summary, we evaluated HPV subtypes in SPs from different regions in a sample of 105 cases. The occurrence of SPs was found to be closely related to HPV infection, especially in the larynx and EAC. Identifying the HPV DNA type can assist in predicting the risk of malignant transformation and assist doctors in determining the prognosis and personalizing follow-ups. However, this study was limited by the small sample size and limited to a single center. Further validation in a larger cohort of samples, with more HPV genotypes, and the inclusion of behaviors associated with HPV infection (such as smoking and sexual activity) is warranted.

## Conclusions

The incidence of HPV has been found to vary significantly depending on the anatomical site, with the larynx exhibiting the highest incidence. HPV infections are associated with the occurrence of SPs in the larynx and EAC, while SPs in the head and neck region are rarely associated with HR-HPV infections. There may be an association between p16 and HPV infection in head and neck SPs, but further studies are needed to validate this assertion that p16 can serve as a supplementary marker of HPV infection in head and neck SPs.

## Supporting information

S1 Dataset (XLSX)
